# Supplementary material for: Visualized in-sensor computing
Source: Nat Commun. 2024 Apr 24;15:3454. doi: 10.1038/s41467-024-47630-9 (PMC11043433; doi:10.1038/s41467-024-47630-9)
Supplement: Supplementary file 1 — Supplementary Information [file 41467_2024_47630_MOESM1_ESM.pdf]

Supplementary Information

Visualized in-sensor computing

Yao Ni[1,2,†], Jiaqi Liu[1,2,†], Hong Han[1,2,†], Qianbo Yu[1,2], Lu Yang[1,2], Zhipeng Xu[1,2], Chengpeng Jiang[1,2], Lu Liu[1,2], Wentao Xu[1,2,*]

Y. Ni, J. Liu, H. Han, Q. Yu, L. Yang, Z. Xu, Dr. C. Jiang, L. Liu, Prof. W. Xu

*Corresponding author. Email: wentao@nankai.edu.cn (Prof. W. Xu).

This Supplementary Information includes:

Supplementary Methods

Supplementary Notes

Supplementary Figs. 1 to 26

Supplementary Tables 1 and 2

Supplementary References 14, 18, 30-35

Supplementary Methods

Image processing and low-frequency suppression filtering simulation

Images were extracted and processed using Python. The RGB was reduced to grayscale as gray = red × 0.299 + green × 0.587 + blue × 0.114. To simulation a low-frequency suppression filter, a grayscale image of a city as an example was first converted from spatial domain to frequency domain by Fourier transform, then the matrix in the frequency domain was rearranged by moving the zero-frequency component to the center. An $m \times n$ filter template was proposed depended on the size of the generating new matrix. A low-frequency suppression filter was established by exploiting the filtering behavior of the ENT low-frequency suppression filter. In the filter template, f is the Euclidean distance between the center (u = 0.5m, v = 0.5n) and a specific point (i, j), as $f = [(i - u)^2 + (j- v)^2]^{0.5}$, and f_c is the cut-off frequency of the filter. The filtering was implemented by utilizing a matrix $G = H(i, j)\ N(i, j)$. G was decentralized and transformed to a matrix in the spatial domain by inverse Fourier transform, to achieve the ultimate result.

Artificial whisker fabrication

First, an equal sandblasting treatment was performed on both sides of the Nafion membrane to improve roughness The Nafion film was sequentially treated in hydrochloric acid (2 mol L^{-1}), deionized water, hydrogen peroxide, and deionized (DI) water for 1 h each process, then immersed in a mixture of 250 mL $Pt(NH_3)_4Cl_2$ (1 wt%) and 20 mL $NH_3{\cdot}H_2O$ (18 wt%) for 24 h. Then the Nafion membrane was transferred into 250 mL DI water, and then 40 mL of 5 wt% $NaBH_4$ as a reducing agent was gradually dropped 20 times over a span of 4 h. Pt walls were prepared on both sides of the Nafion membrane by autocatalytic reduction plating to obtain a bionic actuation layer. The PDMS base was added into the CNT suspension and stirring for 1 h. Then, the curing agent was added into the uniform mixture with a weight ratio of base-to-curing agent at 10: 1 and mixed by magnetic stirring. After degassing in a vacuum oven at room temperature for 2 h, CNT/PDMS composite ink was obtained. The CNT/PDMS

film with a pyramid microstructure was prepared by casting the composite ink onto the micropyramidal side of the mold, and annealed at 100 °C for 1 h. Finally, the CNT/PDMS film was peeled off from mold, assembled with bionic actuation layer, and used as a vibration sensing layer.

Construction of the visualized bionic reflex system

A longicorn beetle specimen was soaked in alcohol for 6 h to soften it. The ENT was attached on back of the beetle. Artificial whiskers were connected to the head of longicorn to form complete bionic receptor and effector. Two signal-processing modules were connected with ENT and the artificial whiskers: the module for afferent nerve converted sampling vibration signal to sampling voltage spikes; the module for efferent nerve amplify the EPSC from ENT units to reach the working threshold of the artificial whiskers.

Supplementary Notes

Supplementary Note 1. *C-V* characteristics

The specific capacitance C can be calculated as

$$C = \frac{1}{2 \cdot s \cdot r \cdot \Delta V} \int_{V_0}^{V_0 + \Delta V} IdV \quad (1)$$

where s is the surface area of the electrode, $r = 300$ mV s^{-1} is the voltage scanning rate, ΔV is the potential drop and V_0 is the lowest voltage in the whole cycle process (Supplementary Fig. 5).

EIS of the ion gel demonstrates the dynamic natures of ion storage and diffusion processes (Figure 1e). The internal resistance R_i is obtained from the intersection of the arc and the horizontal axis; the charge transfer resistance R_{ct} is obtained from the high-frequency semicircle, which represents the process of ions passing through the interface between the electrolyte layer and NML; diffusion velocity of ions in the electrolyte layer is represented by the slope of the linear low-frequency band. The dielectric layer capacitance C_{dl} can be calculated as:

$$C_{dl} = 1/(\omega \cdot R_{ct}) \quad (2)$$

where ω is the angular frequency of the highest point of the arc, and

$$\sigma = L/(R_{ct} \cdot A) \quad (3)$$

σ the ionic conductivity, where L is the thickness of the electrolyte layer, and A is its superficial area.

Supplementary Note 2. XPS survey spectrum analysis

Survey scans of the P3HT/Nafion bilayer film were taken without and with H^+ doping (Supplementary Fig. 6). P3HT exhibits characteristic a photoelectron emission signal at 163.2 eV (S 2*p*); Nafion exhibits characteristic photoelectron emission signals at 169.1 eV (S 2*p*) and 684.8 eV (F 1*s*). Hence, the ratio of the heights of the characteristic photoelectron peaks of P3HT and Nafion provides a measure of the relative concentrations in the surface region of the H^+ doped bilayer. Before doping, the XPS data for bilayer are consistent with a surface composition that is dominated by Nafion (Supplementary Figs. 6a, b). By contrast, the XPS data for the bilayer after

doping is dominated by P3HT, with a corresponding decrease in the Nafion characteristic peaks; this change suggests that the proton doping process is accompanied by Nafion inter-diffusion into the P3HT (Supplementary Figs. 6c, d).

Supplementary Note 3. Time-series neural network architecture

When signal samples are input, electric fields drive anions to accumulate at the upper interface of the channel, where they can generate numerous hole states to increase the electrical conductivity systematically (Supplementary Fig. 13a). When no signal is input, the accumulated anions gradually drift back to equilibrium positions, and a large number of H^+ in the NML have potential shielding effect, so the hole states disappear rapidly, and as a result the channel switches to the inactive state; this change reduces resource use (Supplementary Fig. 13b). After the last input, many of the anions do not diffuse back to their original locations in a short time, so when a subsequent signal is added to the input stream, the remaining anions augment the number of anions that the pulse drives toward the channel interface, so the hole-carrier density increases again in the channel; this process can recall the previous data and strengthen this category by reactivating corresponding nodes (Supplementary Fig. 13c).

Temporal-correlation in neurons is associated with paired-pulse facilitation (PPF), so this functionality is also established in ENT devices. The EPSC is potentiated by two equally-sized spikes with different intervals $0.05 \leqslant \Delta t \leqslant 0.5$ s (Supplementary Fig. 14): The peak of the triggered EPSC was higher for the second spike than for the first spike. The PPF index is defined as the ratio of the amplitudes between the second EPSC (A_2) and the first EPSC (A_1) (Supplementary Fig. 15). The decay of the PPF index with the increase of Δt fits the following double exponential fitting equation that has a rapid decay and a slow decay:

$$\text{PPF index} = 100\% + C_1 \times exp\left(-\frac{\Delta t}{\tau_1}\right) + C_2 \times exp\left(-\frac{\Delta t}{\tau_2}\right) \quad (4)$$

where C_1 and C_2 are respectively the initial facilitation magnitude of the rapid and slow phases; fitted τ_1 = 73 ms and τ_2 = 820 ms represent characteristic relaxation time of the rapid and slow decay. These values are approximately equal to those measured in

biological synapses.

Thus, this temporal-correlation plasticity enables the ENT devices to act as neural units that have a tunable weight that is applicable to time-series signal processing (Supplementary Fig. 16). The ENT exhibites a dynamic state that can switch between inactive and reactivate, and is therefore consistent with the previous GWR characteristic (Supplementary Fig. 12).

Supplementary Note 4. Low-frequency suppression filtering behavior

To quantify the low-frequency suppression filtering behavior of ENT devices, the EPSC was characterized in response to various stimuli that had a range different f (Supplementary Fig. 17). As f increasing, EPSC also increases significantly. The spike-frequency-dependent plasticity (SFDP) index could be fitted using a sigmoidal function; the fitting estimated f_c = 6.37 Hz (Supplementary Fig. 18). To demonstrate the dynamic filtering function of the ENT, an image of a city is used as an example to simulate the filtering process (Supplementary Fig. 19). Compared to the original image, the outline characteristic is sharpened obviously after filtering. Inputs of various frequency led to a stable, repeatable output that changes dynamically in real time (Supplementary Fig. 20).

Supplementary Note 5. Visualized bionic reflex

Considering the persistence of vision, we used the three-frame difference method to process the adjacent 0.2 s- grayscale values and calculated the Euclidean distance $d_{\Delta\mathrm{gray}}$ of the gray difference. If two or more $d_{\Delta\mathrm{gray}}$ values exceeding the visual recognition threshold (> 4) are observed within the visual persistence time (< 0.2 s), they are recorded as observable chromaticity changes. All observable $d_{\Delta\mathrm{gray}}$ values within the visual persistence time range are further weighted as identify weights (Figure 4f), which represents the degree of difficulty that can be effectively observed.

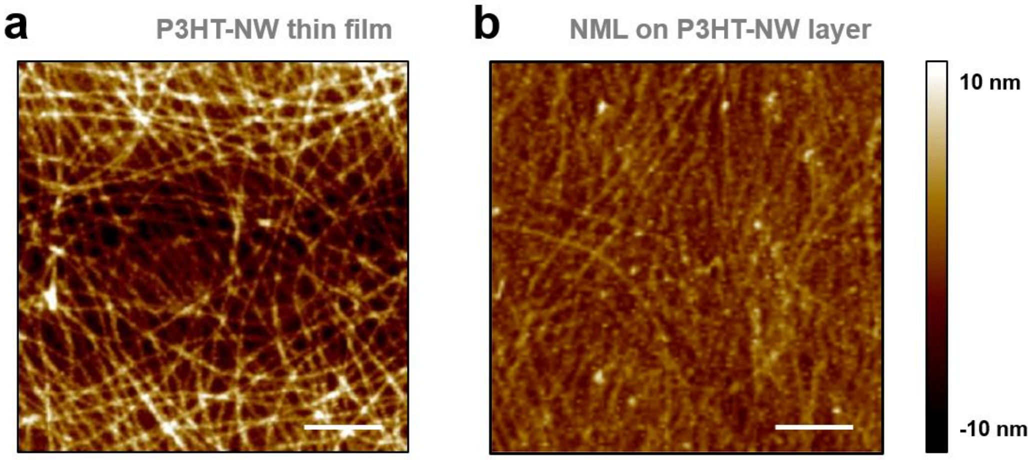

Supplementary Figure 1 | AFM morphologies. Surface morphologies of **a** a P3HT-NW thin film, and **b** a NML on a P3HT-NW thin film. Scale bars, 500 nm.

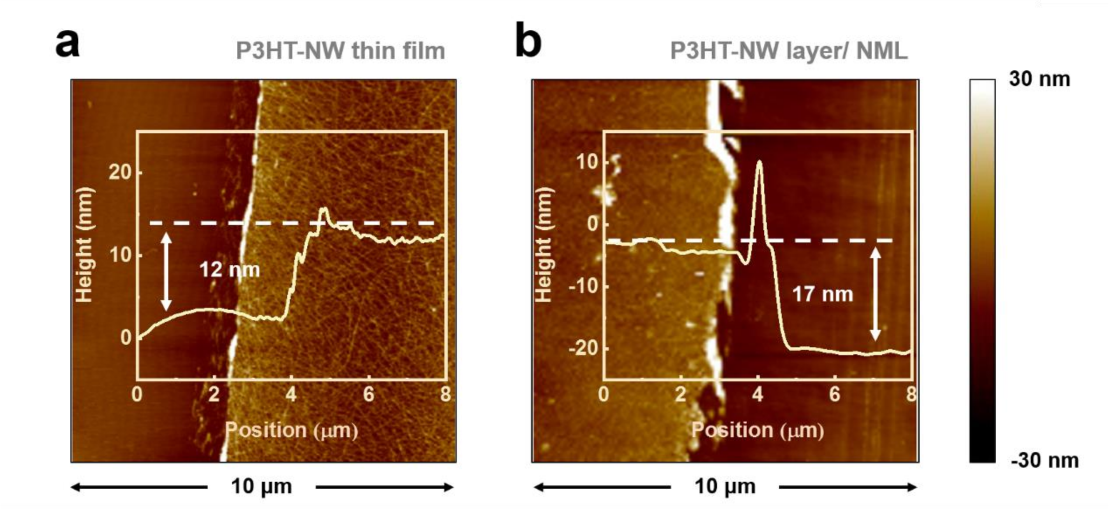

Supplementary Figure 2 | AFM thickness. Cross-sectional profile at the edges of **a** a P3HT-NW thin film and **b** a P3HT-NW layer/NML.

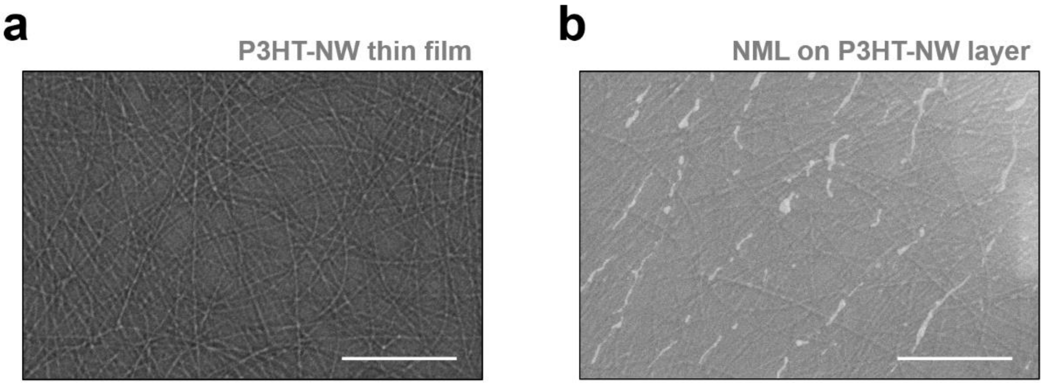

Supplementary Figure 3 | SEM morphologies. SEM morphologies of **a** a P3HT-NW thin film and **b** a NML on a P3HT-NW thin film. Scale bars, 1 μm.

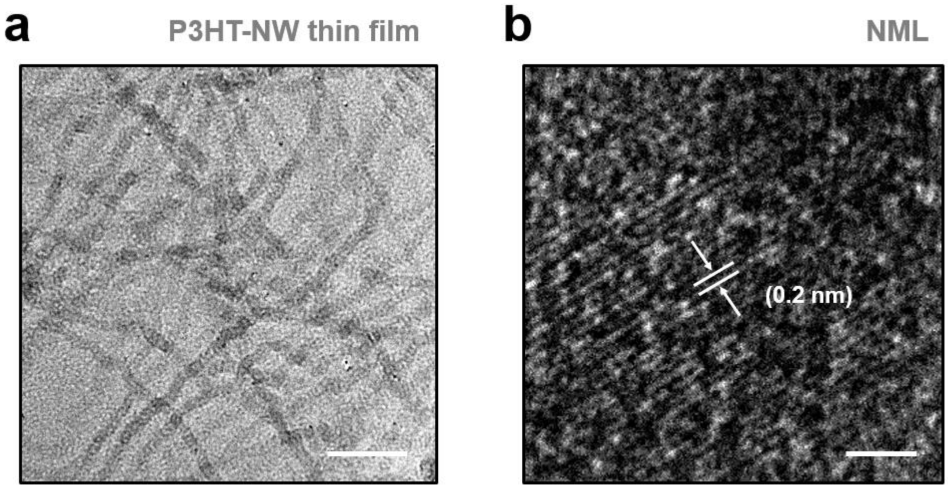

Supplementary Figure 4 | HRTEM morphologies. HRTEM images of **a** a P3HT-NW thin film (Scale bars, 100 nm) and **b** a NML (Scale bars, 1 nm).

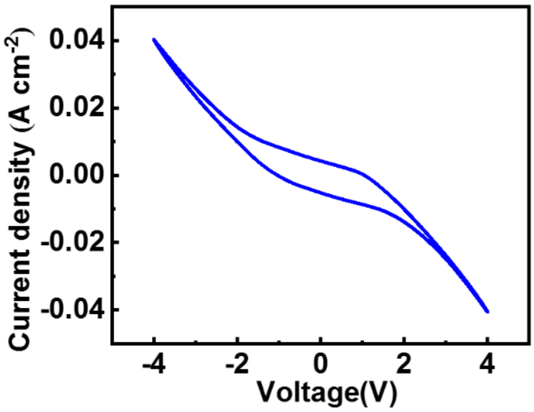

Supplementary Figure 5 | *C*-*V* characteristic curve. Under electrochemical scanning, the change of current density for a diode device of architecture glass/ITO/P3HT/NML/ion gel.

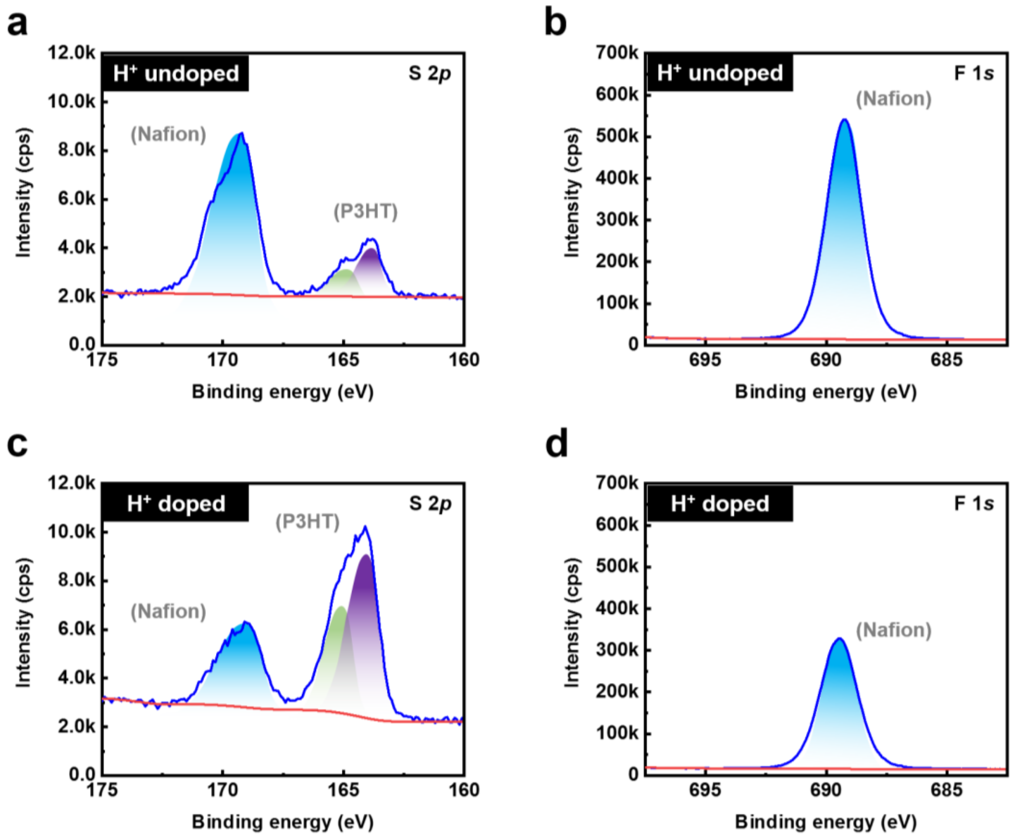

Supplementary Figure 6 | XPS survey spectra. **a** S 2*p* and **b** F 1*s* of P3HT/Nafion film with H^+ undoped. **c** S 2*p* and **d** F 1*s* of P3HT/Nafion film with H^+ doped.

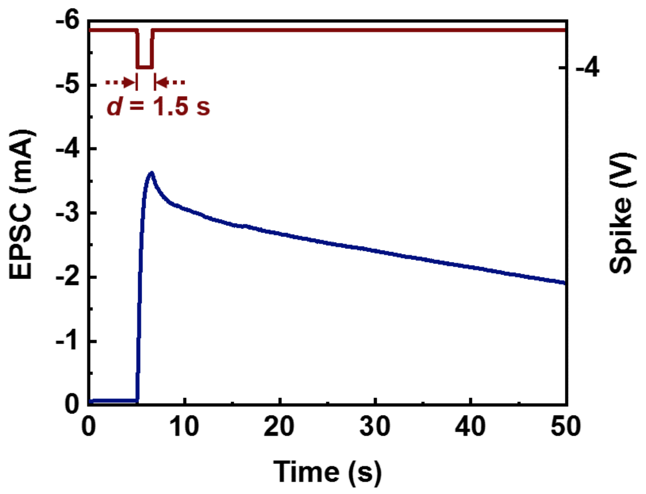

Supplementary Figure 7 | SDDP of P3HT synaptic transistor without Nafion layer. EPSC of P3HT synaptic transistor without Nafion layer triggered by a spike with durations of 1.5 s.

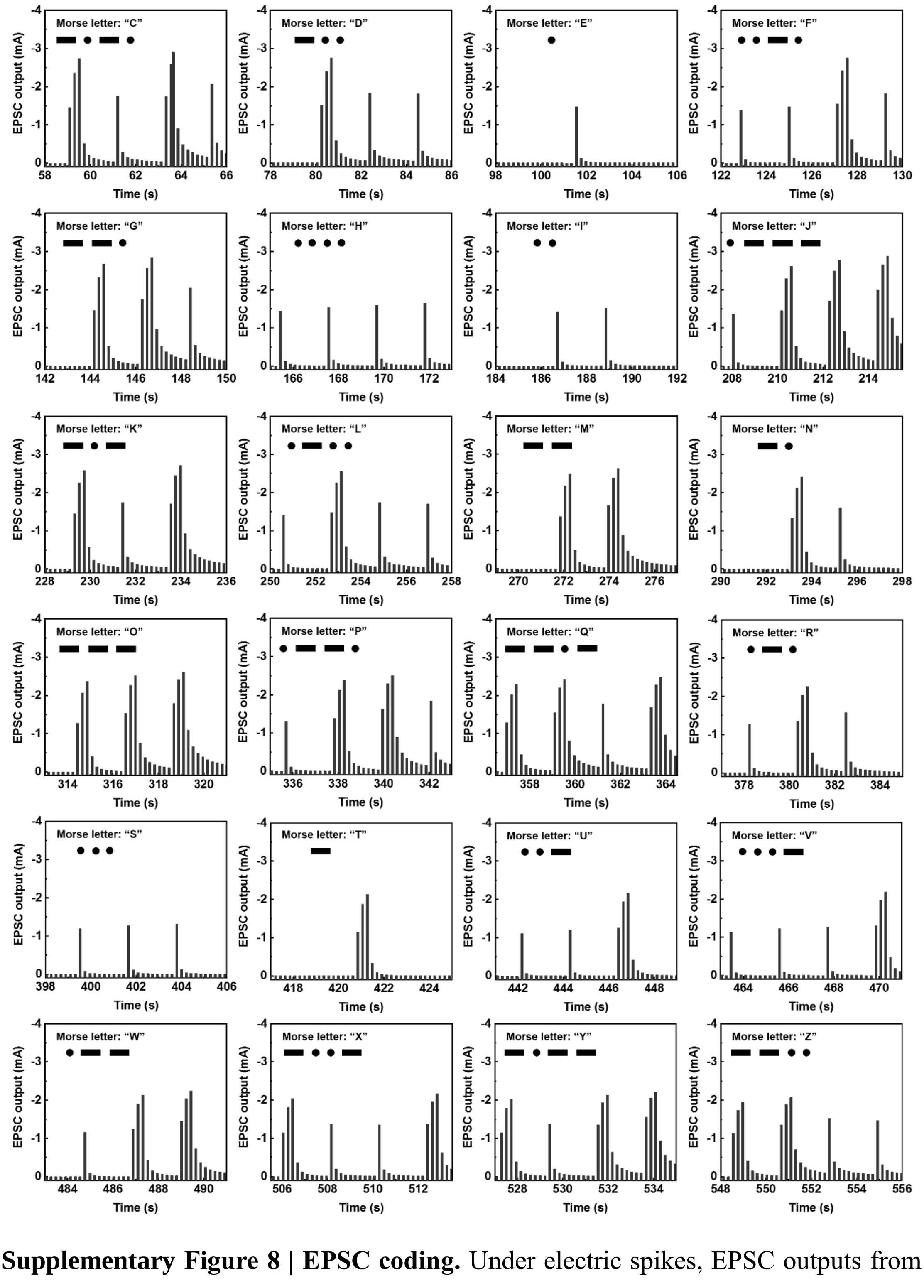

Supplementary Figure 8 | EPSC coding. Under electric spikes, EPSC outputs from ENT representing International Morse code of letters C to Z.

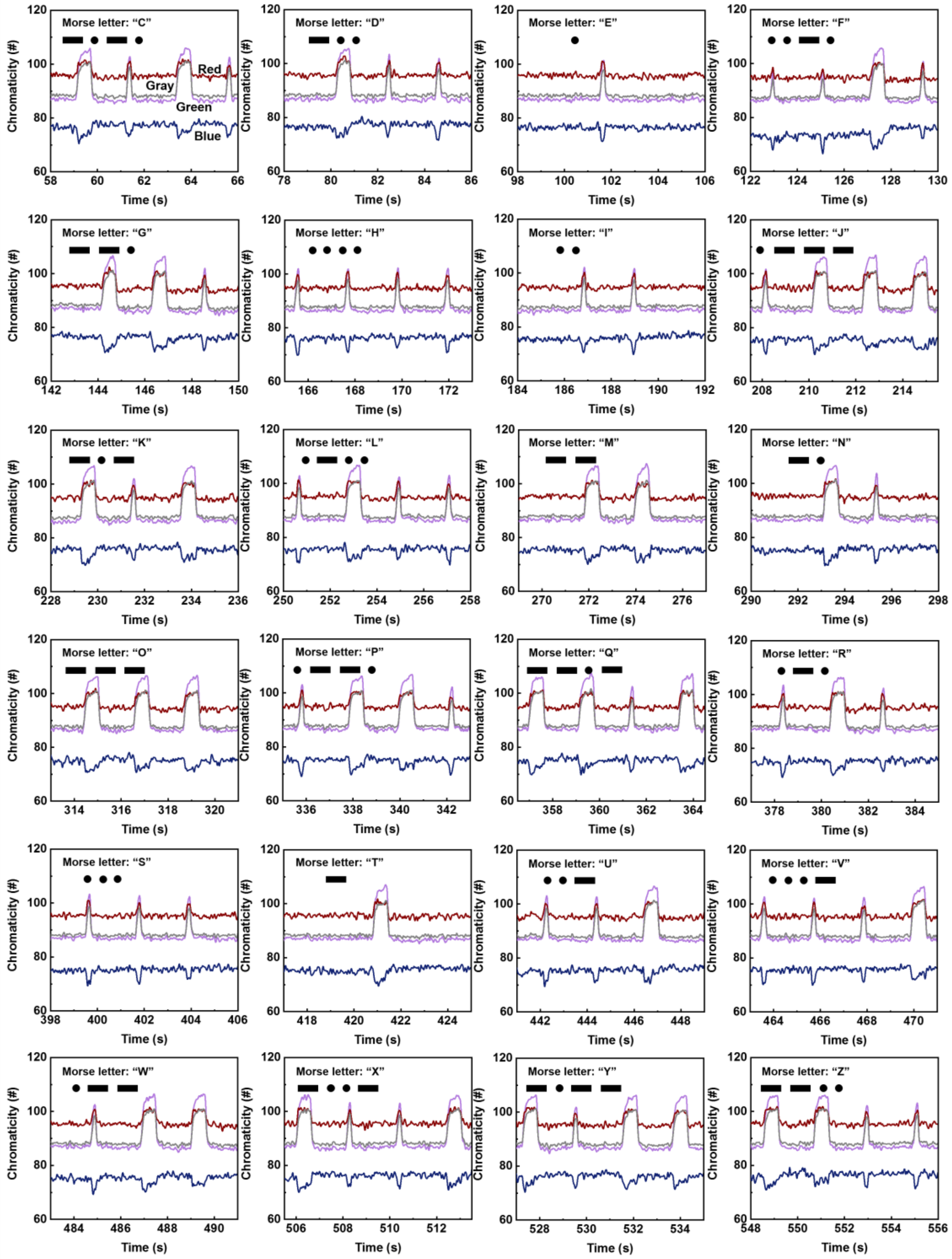

Supplementary Figure 9 | Chromaticity coding. Under electric spikes, chromaticity outputs from ENT, representing International Morse code of letters C to Z.

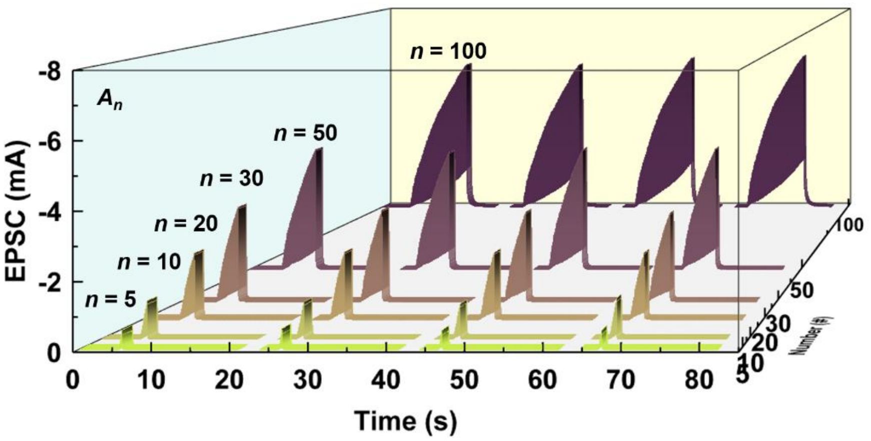

Supplementary Figure 10 | Spike-number-dependent-plasticity. EPSCs triggered by different numbers of spikes from four independent tests of ENT.

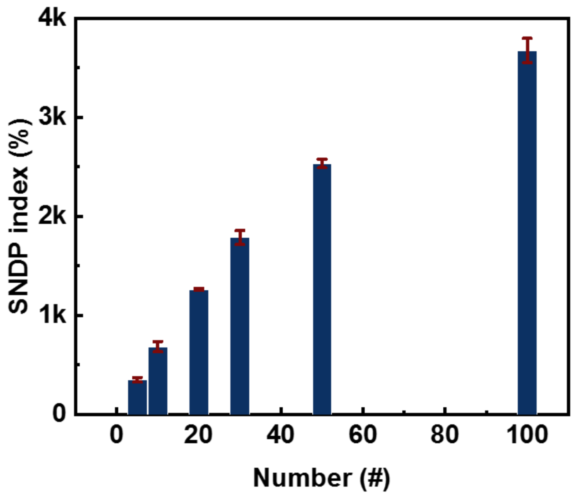

Supplementary Figure 11 | SNDP index. SNDP index ($A_n/A_1 \times 100\%$) according to the number of negative spikes from 5 to 100. The error bars show standard deviations of output value.

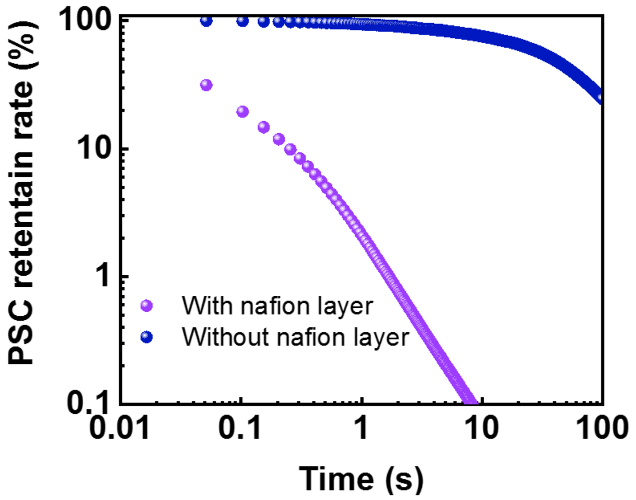

Supplementary Figure 12 | PSC retention rates. PSC retention rates of two types of synaptic transistors after 100 spikes of -4 V.

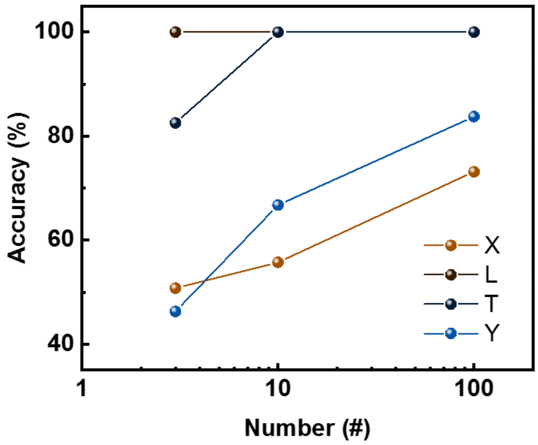

Supplementary Figure 13 | Recognition accuracies of the four classes. With different n_{max}, recognition accuracies of the classes of X, L, T and Y.

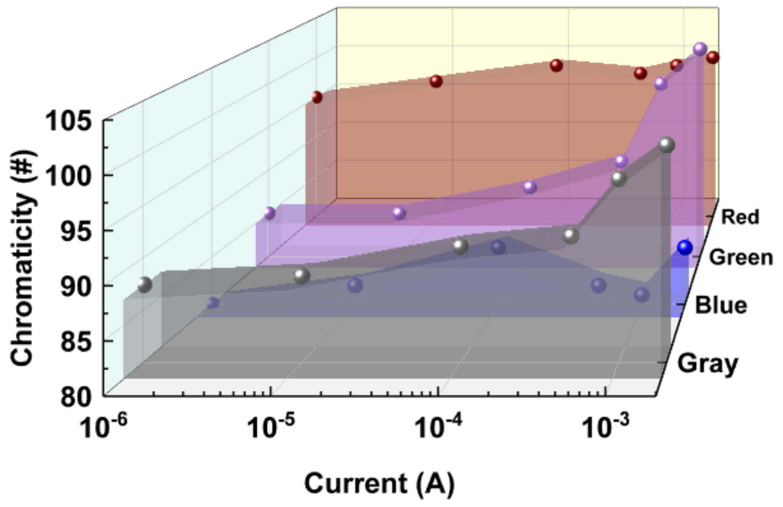

Supplementary Figure 14 | Chromaticity-current map. Chromaticity-current (EPSC) map of the ENT device triggered by spikes of -4 V.

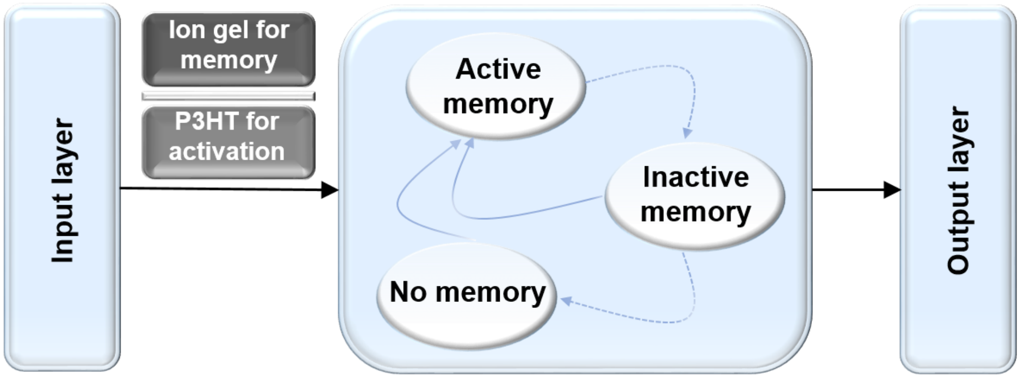

Supplementary Figure 15 | Schematic of a generic TSNN framework. An input layer distributes the signals to a hidden layer, which includes memory and expression functions. Here, the hidden layer is built randomly from programmable ENT devices. The role of the readout layer is to map the dynamics of the hidden layer to the output states.

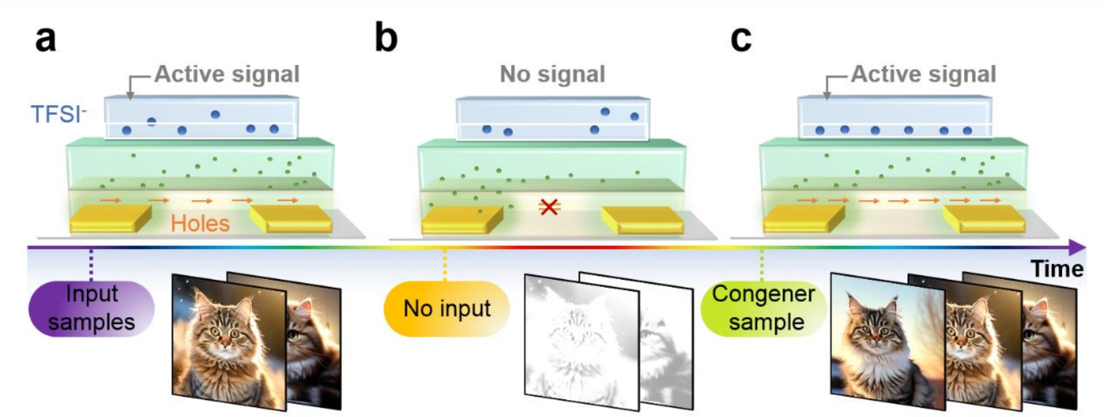

Supplementary Figure 16 | TFSI$^-$ migration and the hole-carrier transfer for TSNN network. a Schematic of the TFSI$^-$ migration in the ion gel and the hole-carrier transfer in the ENT device when signal samples are input to the TSNN network. **b** When no signal appears in the input stream, the corresponding neuron nodes become inactive, so resource usage is reduced. **c** When a signal of the recently-trained category is added to the input stream, the network can recall the previous data and strengthen this category by reactivating corresponding nodes.

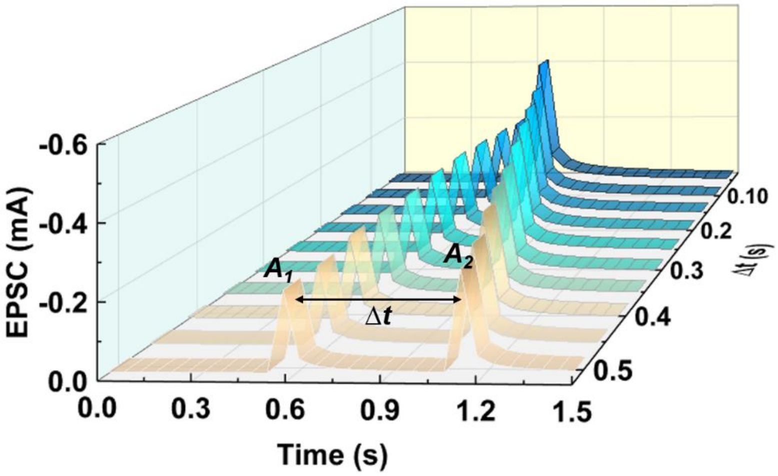

Supplementary Figure 17 | Paired pulse facilitation. Paired EPSC peaks triggered by two equally sized 50 ms spikes with various intervals.

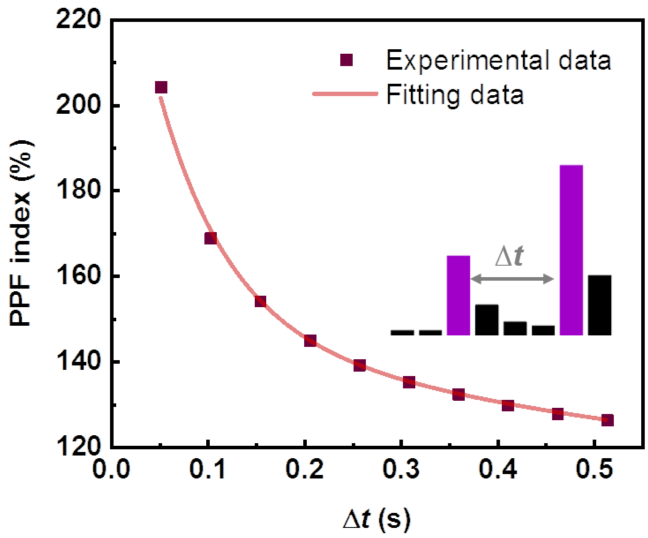

Supplementary Figure 18 | PPF index. PPF index (A_2/A_1 × 100%) versus interval between successive spikes of -4 V.

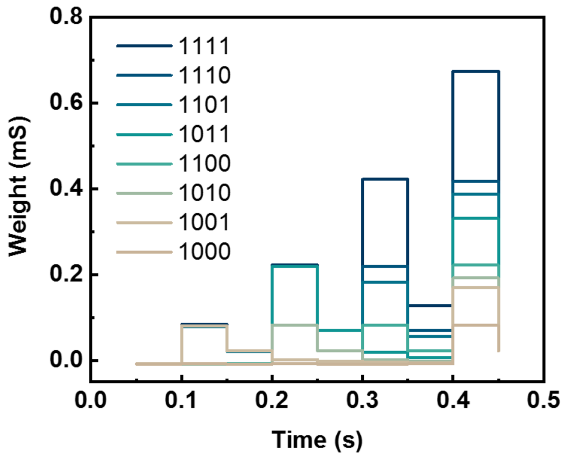

Supplementary Figure 19 | Synaptic weight update of an ENT device. Synaptic weight update triggered by different spike sequences.

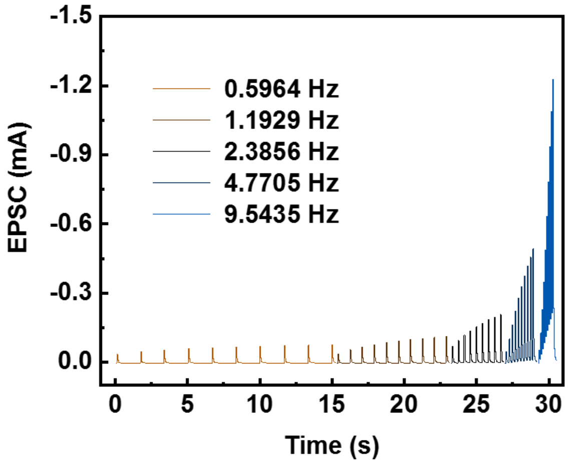

Supplementary Figure 20 | Spike-frequency-dependent plasticity. EPSCs triggered by different frequencies of spikes of -4 V.

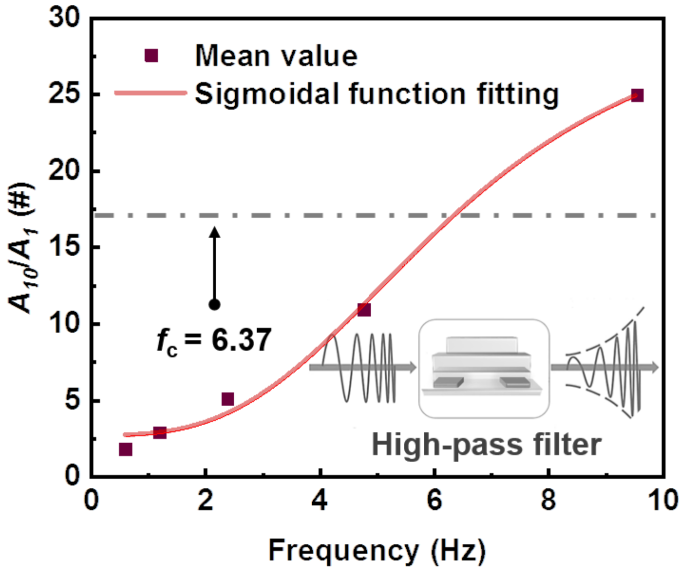

Supplementary Figure 21 | SFDP index. Dependence of the gain of EPSC (A_{10}/A_1) on the pulse frequency; inset: schematic diagram of a low-frequency suppression filtering function.

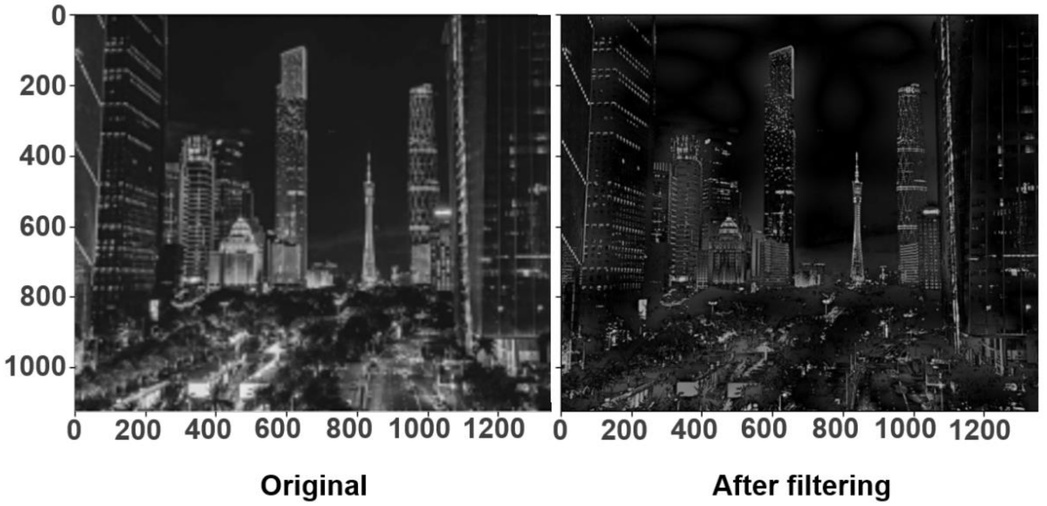

Supplementary Figure 22 | Low-frequency suppression filtering simulation. The original image of a city, and the image of the city after sharpening with the low-frequency suppression filtering function at the cut-off frequency of 6.37 Hz.

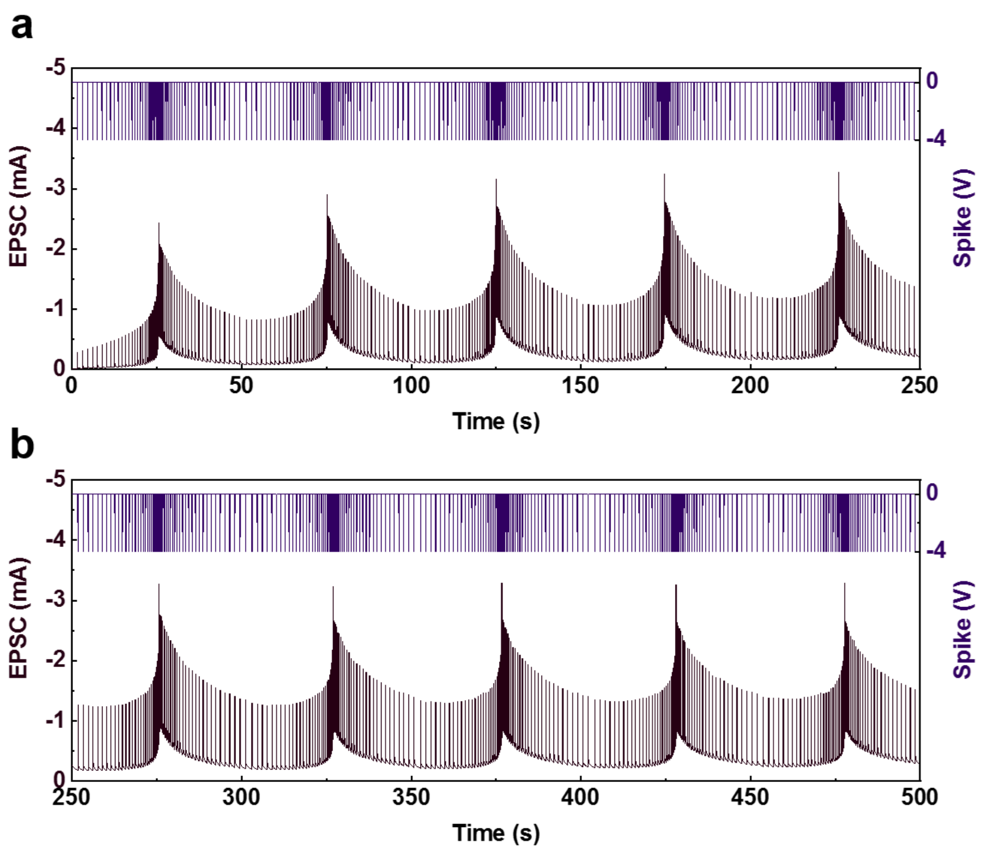

Supplementary Figure 23 | 10 cycles of SFDP. a The first 5 cycles of ENT devices stimulated at spike frequency from 0.6 to 19.5 Hz. **b** The last 5 cycles of ENT devices stimulated at spike frequency from 0.6 to 19.5 Hz.

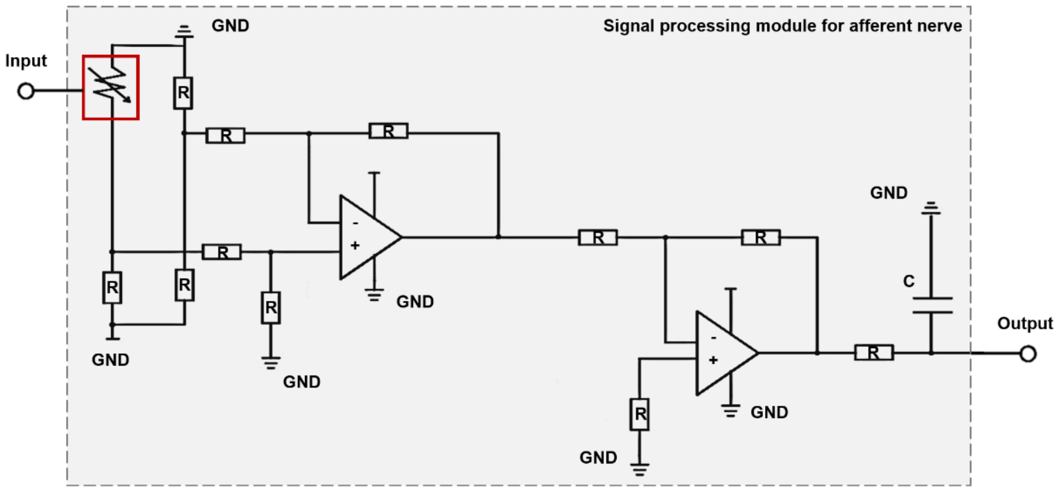

Supplementary Figure 24 | Signal processing module for afferent nerve. Circuit diagrams of the signal-processing module for afferent nerve.

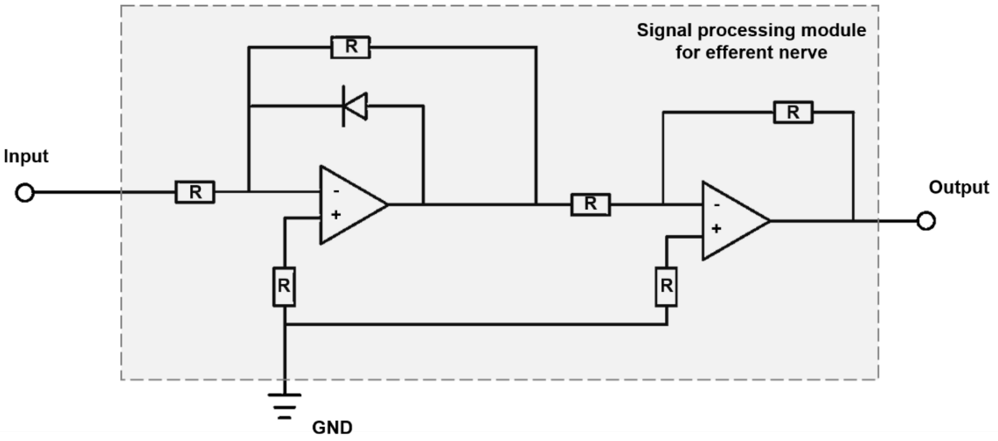

Supplementary Figure 25 | Signal processing module for efferent nerve. Circuit diagrams of the signal-processing module for efferent nerve.

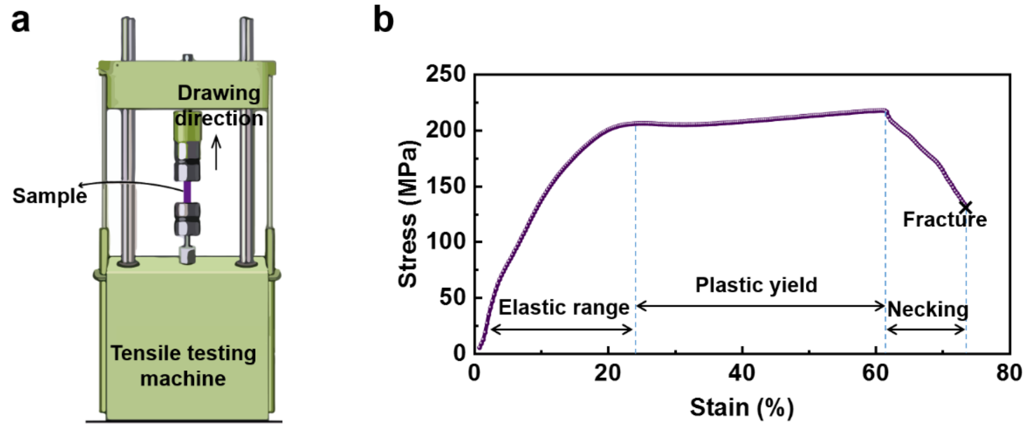

Supplementary Figure 26 | Nominal stress-strain characteristic**. a** Nominal stress-strain testing diagram. **b** Nominal stress-strain curve of ENT.

Supplementary Table 1 Output and input results according to EC, CC, and ECBC modes.

Mode	Input	Dot_1	Dash_1	Dot_2	Dash_2	Dot_3	Dash_3	Dot_4	Dash_4	Output
**EC** (Dot: < 2; Dash: > 2)	D	--	2.75355	1.83728	--	1.8195	--	--	--	D
	L	1.3987	--	--	2.55639	1.73583	--	1.69982	--	L
	G	--	2.67005	--	2.85091	**2.05649**	--	--	--	**O**
	C	--	2.73667	1.75813	--	--	2.91386	**2.07467**	--	**Y**
	Z	--	**1.94315**	--	2.0695	1.53177	--	1.47424	--	**L**
	O	--	2.36805	--	2.51919	--	2.62198	--	--	O
	Y	--	2.01627	1.37151	--	--	2.13128	--	2.21847	Y
**CC** (Dot: < 100; Dash: >100)	D	--	101.45156	**100.55256**	--	99.28089	--	--	--	**G**
	L	98.98778	--	--	101.13956	98.45244	--	98.76656	--	L
	G	--	101.78522	--	101.63611	97.872	--	--	--	G
	C	--	101.28278	99.12756	--	--	101.29467	98.46178	--	C
	Z	--	101.46089	--	101.09411	98.14378	--	99.10589	--	Z
	O	--	101.21533	--	101.35833	--	101.36611	--	--	O
	Y	--	101.61711	97.74278	--	--	101.18733	--	100.81989	Y
**ECBC** (Dot: < 120; Dash: > 120)	D	--	128.98706	118.92536	--	117.47589	--	--	--	D
	L	112.97478	--	--	126.70346	115.81074	--	115.76476	--	L
	G	--	128.48572	--	130.14521	118.4369	--	--	--	G
	C	--	128.64948	116.70886	--	--	130.43327	119.20848	--	C
	Z	--	120.89239	--	121.78911	113.46148	--	113.84829	--	Z
	O	--	124.89583	--	126.55023	--	127.58591	--	--	O
	Y	--	121.77981	111.45788	--	--	122.50013	--	123.00459	Y

Supplementary Table 2 Comparison of recent works relating to artificial neural systems leveraging ion conduction.

Biomimicking concept	Key processing unit leveraging ion conduction	Perception type	Response performance		Year	Ref.
			Actuation	Chrominance-change		
Biological motor system	Optical synaptic device	Photoelectric signal	Strain: 35%	Not given	2019	33
Neurologically integrated soft engineering systems	Rubbery synaptic transistor	Piezoelectric signal	Curvature: 30°	Not given	2019	34
Artificial somatic reflex arc	Threshold controlling unit	Piezoelectric signal	Reversing actuation	Not given	2020	14
Synaptic device amplifier circuit-polymer actuator system	Graphdiyne-based artificial synapse	Electrical signal	Curvature: 20°	Not given	2021	35
High-Strength Neuromuscular System	Parallel-channeled artificial synapse	Electrical signal	Force: 29.2 N; Curvature: 73°	Not given	2022	36
Artificial visual nerve	Perovskite synaptic device	Photoelectric signal	Force: 21.6 N	Not given	2022	37
Low-power stretchable neuromorphic nerve	Stretchable synaptic transistor	Electrophysiological signals	Force: 412 mN Curvature: 40°	Not given	2023	18
Artificially-intelligent cornea	ZTO-fibers artificial synapse	Photoelectric signal	Not given	From light blue to dark blue	2023	38
Visualized bionic reflex system	Electrochromic neuromorphic transistor	Piezoelectric signal	Curvature: 93°	Gray-change: 20		Our work

Supplementary References

14 He, K. *et al.* An artificial somatic reflex arc. *Adv. Mater.* **32**, e1905399 (2020).

18 Lee, Y. *et al.* A low-power stretchable neuromorphic nerve with proprioceptive feedback. *Nat. Biomed. Eng.* (2022).

33 Karbalaei Akbari, M. & Zhuiykov, S. A. bioinspired optoelectronically engineered artificial neurorobotics device with sensorimotor functionalities. *Nat. Commun.* **10**, 3873 (2019).

34 Shim, H. *et al.* Stretchable elastic synaptic transistors for neurologically integrated soft engineering systems. *Sci. Adv.* **5**, eaax4961 (2019).

35 Wei, H. *et al.* Mimicking efferent nerves using a graphdiyne-based artificial synapse with multiple ion diffusion dynamics. *Nat. Commun.* **12**, 1068 (2021).

36 Ni, Y. *et al.* A high-strength neuromuscular system that implements reflexes as controlled by a multiquadrant artificial efferent nerve. *ACS Nano.* **16**, 20294-20304 (2022).

37 Gong, J. *et al.* An artificial visual nerve for mimicking pupil reflex. *Matter.* **5**, 1578-1589 (2022).

38 Qu, S. *et al.* An artificially-intelligent cornea with tactile sensation enables sensory expansion and interaction. *Nat. Commun.* **14**, 7181 (2023).
