# Supplementary material for: Visualized in-sensor computing
Source: Nat Commun. 2024 Apr 24;15:3454. doi: 10.1038/s41467-024-47630-9 (PMC11043433; doi:10.1038/s41467-024-47630-9)
Supplement: Supplementary file 3 — Description of Additional Supplementary Files [file 41467_2024_47630_MOESM3_ESM.pdf]

Description of Additional Supplementary Files

File Name: **Supplementary Movie 1**

Description: Under electric spikes, chromaticity outputs via ENT representing the International Morse code of letters “A”.

File Name: **Supplementary Movie 2**

Description: Under electric spikes, chromaticity outputs via ENT representing the International Morse code of letters “B”.
